# Radiofrequency ablation for liver metastases in patients with gastric cancer as an alternative to hepatic resection

**DOI:** 10.1186/s12885-017-3156-1

**Published:** 2017-03-10

**Authors:** Jin Won Lee, Moon Hyung Choi, Young Joon Lee, Bandar Ali, Han Mo Yoo, Kyo Young Song, Cho Hyun Park

**Affiliations:** 1 Department of Surgery, Seoul St. Mary’s Hospital, College of Medicine, The Catholic University of Korea, Seoul, South Korea; 2 Department of Radiology, Seoul St. Mary’s Hospital, College of Medicine, The Catholic University of Korea, Seoul, South Korea; 3 Present Address: Department of Surgery, Chuncheon Sacred Heart Hospital, College of Medicine, The Hallym University of Korea, Chuncheon, South Korea; 4 Division of Gastrointestinal Surgery, Department of Surgery, Seoul St. Mary’s Hospital, College of Medicine, The Catholic University of Korea, 505 Banpo-dong, Seocho-gu, Seoul 137-701 South Korea

**Keywords:** Gastric cancer, Liver metastases, Radiofrequency ablation

## Abstract

**Background:**

The purpose of this retrospective study was to determine whether RFA could provide an alternative treatment modality for selected patients who are not candidates for hepatic resection.

**Methods:**

A total of 18 consecutive patients with liver metastases alone from gastric cancer treated with radiofrequency ablation (RFA, *n* = 11) or hepatic resection (HR, *n* = 7) at Seoul St. Mary’s Hospital, Korea, between January 2000 and September 2014, were enrolled.

**Results:**

The median OS and DFS in the RFA group were 40.5 ± 22.3 and 10.3 ± 1.07 months, respectively. There was no significant difference between the RFA and HR groups in terms of baseline characteristics except for performance status. Mean survival and DFS times of all patients were 60.1 ± 9.4 and 40.9 ± 10.2 months, respectively. Mean OS times in the HR and RFA groups were 67.5 ± 15.4 and 51.1 ± 9.8 months (*P* = 0.671), respectively, and the mean DFS time in the HR group (74.1 ± 14.2 months) was longer than that in the RFA group (26.9 ± 9.2 months), but the difference was not significant (*P* = 0.076).

**Conclusions:**

In patients who are not candidates for surgical treatment, RFA may be an alternative to HR.

## Background

Gastric cancer (GC) is the second leading cause of cancer-related deaths worldwide [1]. Survival from GC is inversely related to its staging at diagnosis. The liver is the most common site of hematogenous metastases from gastric cancer. Approximately 4–14% of patients with GC develop synchronous or metachronous liver metastases during the course of the disease, and the prognosis for these patients is poor [2–4]. Among them, half of the patients are diagnosed with exclusively hepatic metastases but the others have concurrent extrahepatic disease, such as peritoneal dissemination, extensive lymph node metastases, or direct neoplastic infiltration of adjacent organs [2–6]. Thus, therapeutic decisions in these patients are a challenge for surgeons and oncologists.

Surgical resection, non-surgical ablation techniques, and systemic chemotherapy are options for therapy. Hepatic resection (HR) has been considered to be the most effective treatment for patients with colorectal liver metastases, with a 5-year survival rate of 40–50% [7, 8]. It provides local control of disease, improved disease-free survival (DFS), and better 5-year overall survival (OS) than chemotherapy alone [4]. However, because of aggressive oncological features, limited surgical indications, post-hepatectomy liver failure, and frequent peritoneal dissemination, not all patients with gastric liver metastases are candidates for HR. For example, many patients with gastric liver metastases have accompanying peritoneal dissemination, extensive lymph node metastases, direct invasion of adjacent organs, and metastatic tumors involving multiple segments, which preclude HR at the time of presentation. Thus, various treatments such as systemic chemotherapy, hepatic arterial infusion (HAI) chemotherapy, radiotherapy, and radiofrequency ablation (RFA) have been proposed to improve outcomes [9–11].

RFA has been considered a less invasive therapeutic choice for hepatocellular carcinoma, especially with small tumors (≤3 cm), and has been used increasingly in the treatment of colorectal or gastric liver metastases because of its safety and wide applicability. There have been remarkable developments in RFA techniques for oncological applications [12]. Different retrospective studies have demonstrated that RFA combined with systemic chemotherapy is effective in the treatment of hepatic metastases from GC. However, because of the low number of patients with gastric liver metastases, prospective clinical trials evaluating the long-term outcomes of RFA for liver metastases of GC are still lacking and predicting which patients will benefit from RFA or HR is still unclear.

The purpose of this retrospective study was to determine whether RFA can provide an alternative treatment for selected patients. We compared the long-term results for GC patients with synchronous or metachronous liver metastases, who were treated with RFA or HR. We report our experiences with 18 patients with liver metastases from gastric cancer treated with RFA or HR at our institution.

## Methods

### Patients

The institutional review board of Seoul St. Mary’s Hospital approved the retrospective analysis of anonymous data. The requirement for written informed consent was waived, because the patient records were anonymized and deidentified prior to analysis.

In total, 18 patients with solitary liver metastases from GC, treated with RFA or surgical resection at Seoul St. Mary’s Hospital, Korea, between January 2000 and September 2014, were enrolled. Clinicopathological information was examined retrospectively.

Histological types of primary GC were categorized as differentiated (well-differentiated, moderately differentiated, or papillary) and undifferentiated (signet-ring cell carcinoma, poorly differentiated, or mucinous). All histopathological information was evaluated according to the International Union Against Cancer (UICC) TNM classification (7^th^ edition) [13]. Patients with synchronous hepatic metastases were diagnosed at the time of presentation with GC, on routine staging with computed tomography. Patients with metachronous metastases were considered to be clear of hepatic metastases at the initial curative-intent surgery with R0 resection, but subsequently became symptomatic on follow-up and were diagnosed with hepatic metastases on radiological images.

The feasibility and safety of RFA were discussed with gastric surgeons, medical oncologists, and interventional radiologists. We considered hepatic resection when complete resection (R0) could be achieved successfully and hepatic reservoir function would be preserved after surgery. RFA was considered for patients with unresectable (by any means) disease or high operative risk, such as co-morbidities, poor performance status, and anatomical difficulties that precluded HR or when patients refused surgical treatment. Furthermore, palliative intended RFA was considered for metastatic hepatic lesions > 3 cm in size. Another inclusion criteria was that complete ablation of the metastatic lesion was feasible. Patients with extrahepatic metastases were excluded. The primary endpoints were overall survival (OS) and disease-free survival (DFS).

### Statistical analysis

Clinical outcomes and survival rates in patients treated with RFA and HR were compared using *t*-tests and χ^2^ tests, as appropriate. Statistical analyses were performed using SPSS software (ver. 12.0; SPSS Inc., Chicago, IL). Continuous data were compared using two-tailed Student’s *t*-tests and categorical data were compared using χ^2^ tests. Survival was analyzed using the Kaplan-Meier method and compared using the log-rank test. Overall survival duration was calculated in months from the date of initial RFA or HR to death or last visit to the clinic. Disease-free survival time was calculated in months from the date of RFA (last RFA in cases where patients underwent repeated procedures) or HR to local relapse of tumor, death, or last follow-up. The Cox regression method was used to establish independent predictors for survival and DFS. Multivariate analysis was performed with Cox’s proportional hazard model and factors with *p* values < 0.1 in univariate analyses were included. A *P* value of < 0.05 was considered to indicate statistical significance.

### RFA procedure

All RFA procedures were performed after ultrasound (US) examinations to assess the feasibility of US-guided percutaneous RF ablation. One of two board-certificated radiologists performed all RFA procedures with US guidance using a commercially available system (Radionics, Cool-Tip system; Burlington, MA, USA) and single-needle electrodes with a 2- or 3-cm active tip. Moderate sedation was used with intravenous injections of pethidine hydrochloride (Jeil Pharm. Co., Ltd.), fentanyl citrate (Daihan Pharm. Co., Ltd, Seoul, Korea), or midazolam (Buqwang, Seoul, Korea). Two or more grounding pads were attached to the patient’s legs. The electrode was inserted percutaneously into the lesion and a route to the lesion was monitored using US. The ablation was performed with gradually increased generator output power during 12 min for each lesion. An ablative margin of at least 0.5 cm surrounding the tumor was the therapeutic goal and the achievement of this goal was evaluated by immediate follow-up computed tomography (CT). If residual viable tumor was found on CT, an additional RF ablation was done to achieve a technically successful RFA.

### Follow-up

RFA efficacy was evaluated with a contrast-enhanced CT scan 1 month after RFA. The tumor was considered to exhibit complete necrosis on the basis of two findings: (1) no contrast enhancement was found within the tumor, and (2) the margins of the ablated zone were clear and smooth. In cases where residual tumor was found on the CT scan 1 month after RFA, a repeated procedure was performed until the imaging scan exhibited no contrast enhancement. After confirming complete destruction of metastatic tumors, patients were followed with repeated CT scan every 3 months during the first year and every 6–12 months after the first year.

## Results

**Fig. 1:**
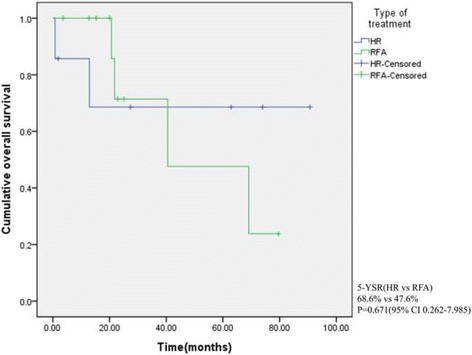
Overall survival of all patients treated with HR and RFA (*P* = 0.671)

**Fig. 2:**
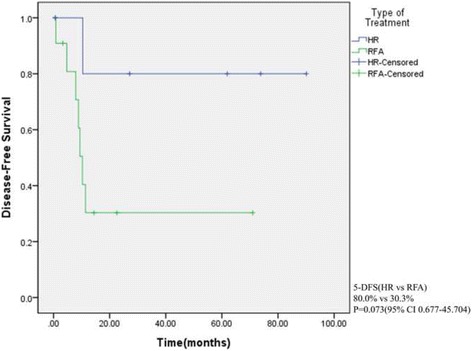
Disease-free survival of all patients treated with HR and RFA (*P* = 0.073)

Between January 2000 and December 2014, 11 patients underwent RFA and 7 underwent HR for synchronous or metachronous liver metastases of GC at our institution. Table 1 summarizes the baseline characteristics of the two groups. All patients received curative resections with D2 lymph node dissection for primary GC. Of the patients, 15 (83.3%) were males and 3 (16.7%) were females. Their median age was 66 years (range, 44–85). There were 6 patients with comorbidities in the RFA group and 2 in the HR group; however, no significant difference was observed between the groups. Regarding performance status, all patients in the HR group had an ECOG score of 0, whereas 5 and 2 patients in the RFA group had ECOG scores of 1 and 2, respectively (*P* = 0.026). The mean survival and DFS times of all patients were 60.15 ± 9.44 and 40.9 ± 10.26 months, respectively. There was no significant difference between the groups in terms of baseline characteristics or tumor-related factors except for systemic chemotherapy after HR or RFA. Systemic chemotherapy after procedures was administered in 87.5% of patients who underwent HR and 36.4% of patients who underwent RFA. The chemotherapeutic regimens included FOLFOX (5-FU, leucovorin, oxaliplatin) and oral agents (tegafur/uracil). Mean overall survival times in the HR and RFA groups were 67.52 ± 15.45 and 51.11 ± 9.87 months, respectively: there was no significant difference in terms of OS between the groups (Fig. 1; *P* = 0.671). The mean DFS times in the HR group (74.16 ± 14.25 months) was longer than that in the RFA group (26.90 ± 9.24 months), but the difference was not significant (Fig. 2; *P* = 0.073).

**Table 1:** Baseline characteristics between HR and RFA groups

Characteristics	HR (*N* = 7) n (%)	RFA (*N* = 11) n (%)	P* value
Sex			
Male	5 (71.4)	10 (90.9)	0.280
Female	2 (28.6)	1 (9.1)	
Age			
<60	2 (28.6)	4 (36.4)	0.732
≥60	5 (71.4)	7 (63.6)	
Comorbidity			
No	5 (71.4)	6 (54.5)	0.474
Yes	2 (28.6)	5 (45.5)	
ECOG PS			
0	7 (100)	4 (36.4)	0.026
1	0 (0)	5 (45.5)	
2	0 (0)	2 (18.2)	
Tumor location			
Upper 1/3	0 (0)	1 (9.1)	0.434
Middle 1/3	2 (28.6)	1 (9.1)	
Lower 1/3	5 (71.4)	9 (81.8)	
Differentation			
Differentiated	4 (51.7)	7 (63.6)	0.783
Undifferentiated	3 (42.9)	4 (36.4)	
Leuren			
Intestinal	3 (42.9)	6 (54.5)	0.629
Diffuse&Mixed	4 (51.7)	5 (45.5)	
Primary Tumor size			
<3 cm	1 (14.3)	1 (9.1)	0.829
≥3 cm	6 (85.7)	10 (90.9)	
Lymphatic invasion			
LI (−)	3 (42.9)	1 (9.1)	0.093
LI (+)	4 (57.1)	10 (90.9)	
Vascular invasion			
VI (−)	4 (57.1)	8 (72.7)	0.494
VI (+)	3 (42.9)	3 (27.3)	
Perineural invasion			
NI (−)	4 (57.1)	8 (72.7)	0.494
NI (+)	3 (42.9)	3 (27.3)	
T stage			
T1-2	1 (14.3)	5 (45.5)	0.171
T3-4	6 (85.7)	6 (54.5)	
N stage			
N0	2 (28.6)	1 (9.1)	0.280
N1-3	5 (71.4)	10 (90.9)	
AJCC 7^th^ stage			
Stage 1–2	3 (42.9)	5 (45.5)	0.914
Stage 3-4	4 (57.1)	6 (54.5)	
Chemotherapy			
CTx (−)	1 (14.3)	1 (9.1)	0.732
CTx (+)	6 (85.7)	10 (90.1)	
Number of metastatic Tumor			
Single	5 (71.4)	8 (72.7)	0.952
Multiple	2 (28.6)	3 (27.3)	
Size of metastatic Tumor			
<3cm	4 (42.9)	6 (54.5)	0.914
≥3cm	3 (57.1)	5 (45.5)	
Lobar distribution			
Unilobar	6 (85.7)	10 (90.9)	0.732
Bilobar	1 (14.3)	1 (9.1)	
Pre-treatment			
Chemotherapy			
CTx (−)	7 (100)	7 (63.6)	0.070
CTx (+)	0 (0)	4 (36.4)	
Post-treatment Chemotherapy			
CTx (−)	1 (14.3)	7 (63.6)	0.040
CTx (+)	6 (85.7)	4 (36.4)	
Complication			
No	5 (71.4)	11 (100)	0.060
Yes	2 (28.6)	0 (0)	

*HR* hepatic resection, *RFA* radiofrequency ablation, *ECOG* Eastern Cooperative Oncology Group; *PS* Performance Status, *LI* lymphatic invasion, *VI* venous invasion, *NI* perineural invasion, *CTx* chemotherapy

*log-rank test

There were 2 patients with postoperative complications (intra-abdominal abscesses) in the HR group, meeting the Clavien-Dindo classification grade IIIa. However, there was no case of complications in the RFA group.

Gender and histological differentiation were independent risk factors for OS in univariate analyses, but neither was associated with overall survival in a multivariate analysis (Table 2). Regarding DFS, univariate log-rank test analysis revealed that vascular invasion of the primary GC and type of treatment were significant prognostic factors (*P* = 0.049), but neither showed a statistically significant difference in a multivariate analysis (Table 3).

**Table 2:** Univariate and multivariate analysis of gastric cancer patients’ clinicopathological features for survival

Characteristics	5-YSR (%)	Univariate *P* value*	HR	Multivariate *P* value
Sex		0.000		0.951
Male	68.2		Ref	
Female	0		408620.765	
Age		0.624		
<60	41.7			
≥60	65.5			
Differentiation		0.034		0.191
Differentiated	85.7		Ref 4.685	
Undifferentiated	25.7			
Primary tumor size		0.853		
<3 cm	66.7			
≥3 cm	58.1			
Lymphatic invasion		0.822		
LI (−)	66.7			
LI (+)	60.2			
Vascular invasion		0.369		
VI (−)	58.2			
VI (+)	50.0			
Perineural invasion		0.959		
PI (−)	67.5			
PI (+)	55.6			
T stage		0.266		
T1-2	40.0			
T3-4	73.3			
N stage		0.448		
N0	50.0			
N1-3	62.2			
AJCC 7^th^ stage		0.779		
Stage 1–2	66.7			
Stage 3-4	56.3			
Number of metastatic Tumor		0.237		
Single	58.6			
Multiple	66.7			
Size of metastatic Tumor		0.427		
<3 cm	64.8			
≥3 cm	58.3			
Lobar distribution		0.821		
Unilobar	66.8			
Bilobar	50.0			
Type of treatment		0.671		
Hepatic resection	68.6			
RFA	47.6			

*5-YSR* 5-year survival rate, *HR* hazard ratio, *LI* lymphatic invasion, *VI* venous invasion, *NI* perineural invasion, *RFA* radiofrequency ablation

*log-rank test

**Table 3:** Univariate and multivariate analysis of gastric cancer patients’ clinicopathological features for DFS

Characteristics	5-DFS (%)	Univariate P value	HR	Multivariate P value
Sex		0.266		
Male	54.2			
Female	0			
Age		0.596		
<60	33.3			
≥60	56.3			
Differentiation		0.932		
Differentiated	50.0			
Undifferentiated	41.7			
Primary tumor size		0.731		
<3 cm	33.3			
≥3 cm	50.3			
Lymphatic invasion		0.731		
LI (−)	33.3			
LI (+)	50.3			
Vascular invasion		0.049		0.061
VI (−)	30.3		Ref 0.128	
VI (+)	80.0			
Perineural invasion		0.858		
PI (−)	50.5			
PI (+)	40.0			
T stage		0.297		
T1-2	33.3			
T3-4	55.6			
N stage		0.761		
N0	50.0			
N1-3	46.4			
AJCC 7^th^ stage		0.227		
Stage 1–2	28.6			
Stage 3-4	63.5			
Number of metastatic Tumor		0.059		
Single	30.3			
Multiple	80.0			
Size of metastatic Tumor		0.702		
<3 cm	40.0			
≥3 cm	62.5			
Lobar distribution		0.683		
Unilobar	46.4			
Bilobar	50.0			
Type of treatment		0.073		0.072
Hepatic resecction	80.0		Ref 7.171	
RFA	30.3			

*DFS* disease-free survival, *HR* hazard ratio, *LI* lymphatic invasion, *VI* venous invasion, *NI* perineural invasion, *RFA* radiofrequency ablation

*log-rank test

### RFA treatment

Table 4 summarizes clinical characteristics and prognostic results. One female patient and 10 of the 11 patients had lymph node metastases. Only Patient 4 was diagnosed with synchronous liver metastases at the time of evaluation for primary GC. He was administered 6 cycles of neoadjuvant chemotherapy (Taxol, Cisplatin) before main treatment and RFA was performed at the time of surgery. Patients 2 and 5 had alcoholic hepatitis and history of cerebrovascular disease, respectively, and their ECOG scores were 1. Patient 8 was diagnosed with toxic hepatitis because of prior chemotherapy. Patient 9 had interstitial lung disease and his ECOG score was 2. Finally, Patient 7 refused surgical treatment for liver metastases because of poor general condition (ECOG score 1) and low tolerance to prior chemotherapy.

**Table 4:** Treatment Outcome of individual patients

Clinical data			Liver metastasis				RFA		Recur					
Case	Comorbidity	PS	No	Lobar	Chronicity	Size (cm)	CTx after RFA	Number of initial RFA	Recur	Pattern	Tx after recur	OS (mon)	DFS (mon)	Survival^a^
1	No	0	1	Uni	Meta	2.6	Yes	2	Yes	Liver	RFA	69.1	10.3	Death
2	Yes	1	1	Uni	Meta	3.7	Yes	1	Yes	Liver	CTx	20.7	0.8	Death
3	No	0	5	Bi	Meta	1	No	2	Yes	Liver	None	40.5	11.4	Death
4	No	1	2	Uni	Syn	2	Yes	1	No			79.6	42.3	Alive
5	Yes	1	1	Uni	Meta	1.7	No	1	Yes	Liver	RFA	21.8	4.7	Death
6	No	0	1	Uni	Meta	3.3	Yes	1	No			22.9	22.6	Alive
7	Yes	1	1	Uni	Meta	0.5	No	1	Yes	Virchow Node	CTx	12.8	9.4	Alive
8	Yes	0	1	Uni	Meta	1.5	No	1	Yes	Lung	None	25.1	8.9	Alive
9	Yes	2	1	Uni	Meta	4.2	No	1	Yes	Liver	None	20.0	7.9	Alive
10	Yes	1	1	Uni	Meta	3.7	No	1	No			3.6	3.3	Alive
11	No	2	2	Uni	Meta	3.2	NO	1	No			15.3	14.4	Alive

*PS*, Performance status based on Eastern Cooperative Oncology Group, *No* number of metastatic lesion; *Lobar*, lobar distributaion; *Uni*, unilobar; *Bi*, bilobar; *Meta*, metachronous; *Syn* synchronous, *CTx* chemotherapy, *Recur* recurrence, *Tx* treatment, *OS* overall survival, *DFS* disease free survival, *mon* months

^a^Survival at the time of analysis

Complete ablation was achieved in 9 patients; 2 patients whose 1 month follow-up CT scans revealed remaining viable tumor underwent repeat ablations and all residual tumor was ablated successfully by the second RFA. Only 4 patients received systemic chemotherapy after RFA, indicating that most patients in the RFA group had poor performance status. Hepatic metastases recurred in 5 patients, lung metastases in 1 patient, and Virchow’s node in 1 patient. Of these, 2 patients underwent RFA again and 2 received second-line systemic chemotherapy. Systemic chemotherapy was administered until disease progression or intolerance of the treatment. Two of the 11 patients survived more than 5 years after initial RFA (Patients 1, 4). Of these, Patient 4 developed a suspicious metastatic pulmonary nodule 41 months after the initial RFA, which was confirmed as an inflammatory nodule by positron emission tomography; it regressed spontaneously. The median OS and DFS were 40.5 ± 22.37 and 10.30 ± 1.07, respectively.

## Discussion

Liver metastasis from GC is generally staged according to the system of the Japanese Gastric Cancer Association (H0, no liver metastasis; H1, liver metastases limited to one lobe of the liver; H2, isolated diverse metastases in both lobes of the liver; H3, multiple distributed metastases in both lobes of the liver) [14]. The prognosis of GC liver metastases is known to be poor with survival of 3–5 months without an effective treatment [15]. For these patients, systemic chemotherapy can be a standard approach and surgical resection has recently been reported to prolong survival in selected patients [16–18].

HR for metastatic tumors has been most recognized treatment option in patients with colorectal cancer, with 5-year survival rates of 37–58% [7]. However, these excellent results with colorectal cancer have not been achieved in gastric cancer, seemingly due to the biological aggressiveness of the disease. Furthermore, not all patients with liver metastases from GC are candidates for surgical resection because of tumor location, functional hepatic reserve after surgery, comorbidities, and synchronous peritoneal dissemination. These patients with unresectable liver metastases have received systemic chemotherapy; however, the results have been disappointing.

Recently, given the disappointing prognosis of ‘conventional’ systemic chemotherapy for GC with liver metastases, RFA has been regarded as an alternative to HR for treating primary or metastatic tumors in selected patients [19, 20]. Several groups have reported the benefit of RFA in treating hepatic metastases from GC [6, 9, 11, 21]. Kim et al. [10] addressed the rationale for RFA, suggesting that cytoreductive procedures enable chemotherapy to be more effective and that removal of an isolated metastatic deposit can prevent further dissemination of the disease to other sites. Dittmar et al. [6] concluded that RFA may be a useful alternative in patients where surgery is not feasible in a retrospective study with 15 patients who underwent liver resection or RFA for hepatic metastases from GC. Furthermore, Chen et al. [9] argued that patients with solitary liver metastases benefit from RFA, which is minimally invasive and considered a safe modality. However, few studies have compared prognosis after treatment of GC liver metastases with RFA or HR, and the available results have been conflicting. Hwang et al. [22] considered 72 patients with metachronous metastases subjected to different treatments, but not to hepatectomy. Of their cohort, the 15 patients without extrahepatic disease treated by RFA combined with systemic chemotherapy had a median survival of 22 months, with 3- and 5-year survival rates of 50 and 40%, respectively, similar to those reported in the best surgical series. Given this background, we performed RFA in patients with liver metastases from GC and compared the results with those of HR.

RFA is a less invasive procedure than surgical resection and can be performed repeatedly in case of incomplete ablation or recurrent tumors safely. In our study, 2 patients underwent repeated RFA for remnant viable tumors. However, several studies have noted that RFA was associated with a higher recurrence rate. Those studies reported that tumors > 3 cm in diameter were associated with local recurrence after RFA [23–25].

In our cohort, we found that the mean DFS in the HR group was longer than that in the RFA group (74.16 ± 14.25 vs. 26.90 ± 9.24; *P* = 0.073; Fig. 2). The mean survival times were 64.52 ± 15.45 and 51.11 ± 9.87 months for those patients who underwent HR or RFA for liver metastases, respectively; there was no significant difference in OS. From this perspective, although HR was superior to RFA in terms of DFS, RFA could be an alternative to HR, especially in patients who have comorbidities or borderline resectability because of the less invasive nature and repeatability of the procedure. In our cases, 2 patients lived more than 5 years after procedures, and one of them (Patient 1 in Table 3) underwent repeated RFA for incomplete ablation.

Recently, several factors, such as the number of metastatic tumors, maximal size, status of lobar distribution, chronicity, and combination with systemic chemotherapy, were identified to be independent prognostic factors after treatment of liver metastases from GC [26, 27]. However, in our multivariate analysis, none of these factors was associated with OS or DFS. Therefore, we believe that this procedure can be applied to carefully selected patients, even from a point of oncological view.

This study has several limitations including its retrospective nature and small sample size. Moreover, 3 patients in the HR group were lost to follow-up during the study period, so we were unable to collect information about their survival or disease relapse. Additionally, systemic chemotherapy after HR or RFA was not uniform during the study period. Finally, the criteria used to select treatment options at the time of diagnosis were not documented and were subject to individual physician decisions, which might have caused inevitable selection bias. In the future, a large-scale, well-controlled, prospective study is needed to compare efficacy between RFA and HR for patients with GC with liver metastases.

Despite these limitations, our results showed that the RFA was not inferior to HR in terms of OS and can be considered as a treatment option in selected patients. In fact, for patients whose general condition often contraindicates surgery, a less invasive ablative technique may represent an interesting opportunity.

## Conclusions

Hepatic resection may be the optimal treatment option for gastric liver metastases. However, in patients who are not candidates for surgical treatment (e.g., old age, comorbidities, poor general condition, bilobar distribution of metastatic tumors, patient refusal), radiofrequency ablation could be an alternative to hepatic resection. It is a less invasive treatment option for liver metastases alone from gastric cancer and offers these patients non-inferior survival outcome to hepatic resection despite a high recurrence rate.

## Abbreviations

CT: Computed tomography
DFS: Disease-free survival
ECOG: Eastern Cooperative Oncology Group
GC: Gastric cancer
HAI: Hepatic arterial infusion
HR: Hepatic resection
OS: Overall survival
RFA: Radiofrequency ablation
US: Ultrasound

## References

[1] Siegel R, Naishadham D, Jemal A (2013). Cancer statistics, 2013. CA Cancer J Clin.

[2] Dicken BJ, Bigam DL, Cass C (2005). Gastric adenocarcinoma: review and considerations for future directions. Ann Surg.

[3] D’Angelica M, Gonen M, Brennan MF (2004). Patterns of initial recurrence in completely resected gastric adenocarcinoma. Ann Surg.

[4] Hwang J-E, Kim S-H, Jin J (2014). Combination of percutaneous radiofrequency ablation and systemic chemotherapy are effective treatment modalities for metachronous liver metastases from gastric cancer. Clin Exp Metastasis.

[5] Sakamoto Y, Ohyama S, Yamamoto J (2003). Surgical resection of liver metastases of gastric cancer: an analysis of a 17-year experience with 22 patients. Surgery.

[6] Dittmar Y, Altendorf-Hofmann A, Rauchfuss F (2012). Resection of liver metastases is beneficial in patients with gastric cancer: report on 15 cases and review of literature. Gastric Cancer Off J Int Gastric Cancer Assoc Jpn Gastric Cancer Assoc.

[7] Hur H, Ko YT, Min BS (2009). Comparative study of resection and radiofrequency ablation in the treatment of solitary colorectal liver metastases. Am J Surg.

[8] Grimes N, Devlin J, Dunne DFJ (2014). The role of hepatectomy in the management of metastatic gastric adenocarcinoma: a systematic review. Surg Oncol.

[9] Chen J, Tang Z, Dong X (2013). Radiofrequency ablation for liver metastasis from gastric cancer. Eur J Surg Oncol J Eur Soc Surg Oncol Br Assoc Surg Oncol.

[10] Kim HR, Cheon SH, Lee K-H (2010). Efficacy and feasibility of radiofrequency ablation for liver metastases from gastric adenocarcinoma. Int J Hyperth Off J Eur Soc Hyperthermic Oncol North Am Hyperth Group.

[11] Kim HO, Hwang SI, Hong HP (2009). Radiofrequency ablation for metachronous hepatic metastases from gastric cancer. Surg Laparosc Endosc Percutan Tech.

[12] Ahmad A, Chen SL, Kavanagh MA (2006). Radiofrequency ablation of hepatic metastases from colorectal cancer: are newer generation probes better?. Am Surg.

[13] Edge SB, Compton CC (2010). The American Joint Committee on Cancer: the 7th edition of the AJCC cancer staging manual and the future of TNM. Ann Surg Oncol.

[14] Japanese Gastric Cancer Association (1998). Japanese Classification of Gastric Carcinoma, 2nd English Edition. Gastric Cancer Off J Int Gastric Cancer Assoc Jpn Gastric Cancer Assoc.

[15] Jerraya H, Saidani A, Khalfallah M (2013). Management of liver metastases from gastric carcinoma: where is the evidence?. Tunis Med.

[16] Cheon SH, Rha SY, Jeung H-C (2008). Survival benefit of combined curative resection of the stomach (D2 resection) and liver in gastric cancer patients with liver metastases. Ann Oncol Off J Eur Soc Med Oncol ESMO.

[17] Sakamoto Y, Sano T, Shimada K (2007). Favorable indications for hepatectomy in patients with liver metastasis from gastric cancer. J Surg Oncol.

[18] Takemura N, Saiura A, Koga R (2012). Long-term outcomes after surgical resection for gastric cancer liver metastasis: an analysis of 64 macroscopically complete resections. Langenbecks Arch Surg Dtsch Ges Fur Chir.

[19] Siperstein AE, Berber E, Ballem N (2007). Survival after radiofrequency ablation of colorectal liver metastases: 10-year experience. Ann Surg.

[20] Ni J, Xu L, Sun H (2013). Percutaneous ablation therapy versus surgical resection in the treatment for early-stage hepatocellular carcinoma: a meta-analysis of 21,494 patients. J Cancer Res Clin Oncol.

[21] An JY, Kim JY, Choi MG (2008). Radiofrequency ablation for hepatic metastasis from gastric adenocarcinoma. Yonsei Med J.

[22] Hwang S-E, Yang D-H, Kim C-Y (2009). Prognostic factors for survival in patients with hepatic recurrence after curative resection of gastric cancer. World J Surg.

[23] Elias D, Baton O, Sideris L (2004). Local recurrences after intraoperative radiofrequency ablation of liver metastases: a comparative study with anatomic and wedge resections. Ann Surg Oncol.

[24] Siperstein A, Garland A, Engle K (2000). Local recurrence after laparoscopic radiofrequency thermal ablation of hepatic tumors. Ann Surg Oncol.

[25] Bowles BJ, Machi J, Limm WM (2001). Safety and efficacy of radiofrequency thermal ablation in advanced liver tumors. Arch Surg Chic Ill.

[26] Zacherl J, Zacherl M, Scheuba C (2002). Analysis of hepatic resection of metastasis originating from gastric adenocarcinoma. J Gastrointest Surg Off J Soc Surg Aliment Tract.

[27] Roh HR, Suh K-S, Lee H-J (2005). Outcome of hepatic resection for metastatic gastric cancer. Am Surg.

